# Phytochemical analysis and in vitro anti-proliferative activity of *Viscum album* ethanolic extracts

**DOI:** 10.1186/s12906-020-02987-4

**Published:** 2020-07-09

**Authors:** Carla Holandino, Michelle Nonato de Oliveira Melo, Adriana Passos Oliveira, João Vitor da Costa Batista, Marcia Alves Marques Capella, Rafael Garrett, Mirio Grazi, Hartmut Ramm, Claudia Dalla Torre, Gerhard Schaller, Konrad Urech, Ulrike Weissenstein, Stephan Baumgartner

**Affiliations:** 1 Multidisciplinary Laboratory of Pharmaceutical Sciences, Faculty of Pharmacy, Federal University of Rio de Janeiro, Rio de Janeiro, Brazil; 2 Hiscia Institute, Society for Cancer Research, Arlesheim, Switzerland; 3 Metabolomics Laboratory, Chemistry Institute, Federal University of Rio de Janeiro, Rio de Janeiro, Brazil; 4 Biophysics Institute, Federal University of Rio de Janeiro, Rio de Janeiro, Brazil; 5 Institute of Complementary and Integrative Medicine, University of Bern, Bern, Switzerland; 6 Institute for Integrative Medicine, University of Witten/Herdecke, Herdecke, Germany

**Keywords:** Mistletoe, *Viscum album* L., Phytochemistry, Anti-proliferative activity, Cytotoxicity

## Abstract

**Background:**

*Viscum album* L. (Santalaceae), commonly known as mistletoe, is a hemiparasitic plant traditionally used in complementary cancer treatment. Its antitumor potential is mostly attributed to the presence of aqueous soluble metabolites; however, the use of ethanol as solvent also permits the extraction of pharmacological compounds with antitumor potential. The clinical efficacy of mistletoe therapy inspired the present work, which focuses on ethanolic extracts (*V. album* “mother tinctures”, MT) prepared from different host trees.

**Methods:**

Samples from three European subspecies (*album, austriacum,* and *abietis*) were harvested, and five different *V. album*-MT strains were prepared. The following phytochemical analyses were performed: thin layer chromatography (TLC), high-performance liquid chromatography (HPLC) and liquid chromatography-high resolution mass spectrometry (LC-HRMS). The proliferation assay was performed with WST-1 after incubation of tumor (Yoshida and Molt-4) and fibroblast cell lines (NIH/3 T3) with different MT concentrations (0.5 to 0.05% v/v). The cell death mechanism was investigated by flow cytometry (FACS) using Annexin V-7AAD.

**Results:**

Chemical analyses of MT showed the presence of phenolic acids, flavonoids and lignans. The MT flavonoid and viscotoxin contents (mg/g fresh weight) were highest in *Quercus robur* (9.67 ± 0.85 mg/g) and *Malus domestica* (3.95 ± 0.58 mg/mg), respectively. The viscotoxin isoform proportions (% total) were also different among the VA subspecies with a higher content of A3 in *V. album* growing on *Abies alba* (60.57 ± 2.13). The phytochemical compounds as well as the viscotoxin contents are probably related to the antitumor effects of MT. The cell death mechanisms evaluated by colorimetric and FACS methodologies involved necrotic damage, which was host tree-, time- and dose- dependent, with different selectivity to tumor cells. Mother tincture from *V. album* ssp. *abietis* was the most effective at inducing in vitro cellular effects, even when incubated at the smallest concentration tested, probably because of the higher content of VT A3.

**Conclusion:**

Our results indicate the promising antitumor potential of *Viscum album* ethanolic extracts and the importance of botanical and phytochemical characterization for in vitro anti-proliferative effects.

## Background

*Viscum album* L. (Santalaceae), also known as European mistletoe, is a hemiparasitic shrub that is differentiated into 3 main subspecies growing on different host trees, i.e., *V. album* ssp. *album*, deciduous trees; *V. album* ssp. *abietis*, fir, and *V. album* ssp. *austriacum*, mainly on pine [1].

Most investigations into *V. album* are based on aqueous extracts [2–6]. The active compounds that have been identified are proteins commonly classified as viscotoxins (VT) and mistletoe lectins [7, 8]. Phytochemical investigations of *V. album* also revealed the presence of other important pharmacological compounds, such as phenolic acids, phenylpropanoids, flavonoids, triterpenes, phytosterols, oligopeptides and polysaccharides [9, 10].

The European Medicines Agency reported the traditional use of different *V. album* ethanolic extracts to treat cardiovascular disease [11]. Additionally, Poruthukaren et al. [12] described a reduction in blood pressure after *V. album* ethanolic use for 12 weeks.

Nevertheless, the antitumor effects of ethanolic *V. album* extracts in biological systems have been described. In vivo studies have shown the anticancer activity of alcoholic and glycerine *V. album* extracts by stimulating immune mechanisms and inhibiting tumor cell proliferation [13]. The simultaneous application of *V. album* ethanolic extract and doxorubicin increases the toxicity in Ehrlich tumor cells, opening up the possibility of exploring a tentative synergy between single chemical compounds and complex herbal extracts [14, 15]. In addition, the in vitro research performed with these extracts highlighted the apoptotic mechanisms and cell growth reduction [16, 17] involved in melanoma, leukemia [16], and human cervix adenocarcinoma tumor [17] death. Panossian et al. [18] suggested that phenylpropanoids detected in *V. album* ethanolic extracts had antitumor activity through the inhibition of protein kinase C.

Furthermore, a recent study performed with *V. album* ethanolic extracts showed tumor cell cycle arrest and apoptotic death in in vitro models. Chemical analysis of these extracts identified compounds such as caffeic acid, chlorogenic acid, sakuranetin, isosakuranetin, syringenin 4-O-glucoside, syringenin 4-O-apiosyl-glucoside, alangilignoside C and ligalbumoside A [16].

It is widely known that many metabolites isolated from European mistletoe are not produced by the plant itself but are due to host tree metabolism [19], supporting the importance of understanding the subspecies of European mistletoe and the influence of the host tree. This work shows, for the first time, the in vitro anti-proliferative effects and the chemical composition of ethanolic extracts produced from different *V. album* host trees.

## Methods

### Plant growth and harvest

**Fig. 1:**
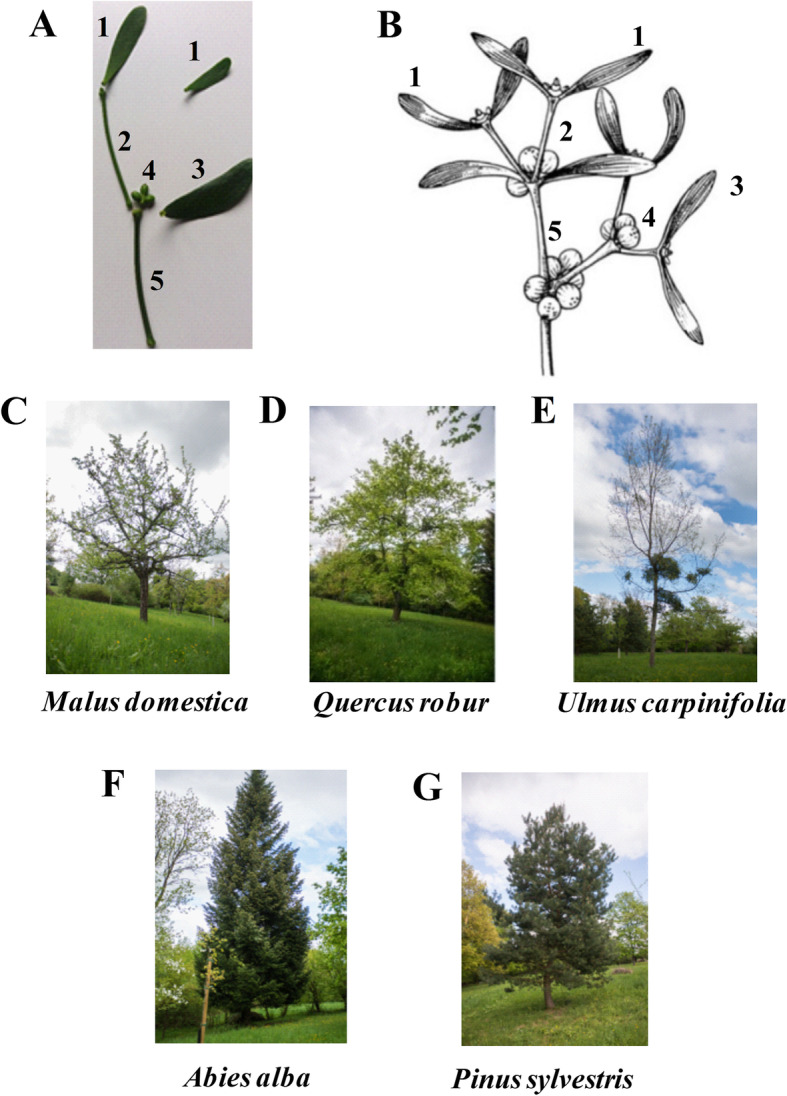
*V. album* L. representative images describing plant parts used in the ethanolic extracts (**a**-**b**) and the different host trees harvested (**c**-**g**). **a** Original picture of a *Viscum album* bush. ^1^First youngest leaves, ^2^first youngest stems, ^3^second youngest leaves, ^4^green unripe berries, ^5^second youngest stems; **b***Viscum album* schematic figure. ^1^First youngest leaves, ^2^first youngest stems, ^3^ second youngest leaves, ^4^unripe berries, ^5^second youngest stems. **c***V. album* ssp. *album* growing on *Malus domestica*; **d***V. album* ssp. *album* growing on *Quercus robur*; **e***V. album* ssp. *album* growing on *Ulmus carpinifolia*; **f***V. album* ssp. *abietis* growing on *Abies alba*; **g***V. album* ssp. *austriacum* growing on *Pinus sylvestris*

The green, unripe berries, leaves and stems of three European subspecies of *V. album* L. were harvested in July 2016 in natural habitats in Switzerland (Fig. 1a-g). The following *V. album* subspecies were collected from five different host trees: *V. album* ssp. *album* growing on *Malus domestica* (VAM; Fig. 1c), *Quercus robur* (VAQ; Fig. 1d) and *Ulmus carpinifolia* (VAU; Fig. 1e); *V. album* ssp. *abietis* growing on *Abies alba* (VAA; Fig. 1f); and *V. album* ssp. *austriacum* growing on *Pinus sylvestris* (VAP; Fig. 1g). From each host tree, at least five bushes of the same age containing the same parts of the plant were submitted to solvent extraction as follows: first and second youngest leaves, berries, and first and second youngest stems (Fig. 1a-b) were harvested five times. The samples were always collected in the morning (from 8:00 to 11:30 am) and immediately transported to perform ethanolic extraction within 4 to 6 h.

All plants were identified by Dr. Marcelo Guerra Santes (Universidade Estadual do Rio de Janeiro). Voucher specimens (C.H. Quaresma 18.328, C.H. Quaresma 18.329, C.H. Quaresma 18.332, C.H. Quaresma 18.327 and C.H. Quaresma 18.331) were deposited at the Herbarium of the Faculdade de Formação de Professores, Universidade Estadual do Rio de Janeiro, Brazil.

### Preparation of *V. album* mother tinctures

All solvents and reagents exhibited analytical purity quality. The fresh material (5 g) was fragmented into segments smaller than 5 cm long and dried in an oven at 105 °C for 2 h, following the Brazilian Homeopathic Pharmacopeia [20] and the French Pharmacopeia [21]. Once the percentage of solid residue of each fresh plant had been established, the total volume of mother tincture (MT), as well as the volume and the concentration of ethanol used in the maceration process, was determined. Next, the maceration extractive process was conducted over a period of 3 weeks at room temperature in 80% w/w ethanol. To increase the efficiency of the extraction process, all *V. album* ethanolic solutions were shaken by hand for 60 s twice a day. After 3 weeks, the macerates were filtered and kept at 20 ± 5 °C. The final MT concentrations were 40 to 50% v/v [21].

### Thin layer chromatography

Thin layer chromatography (TLC) was performed as specified in the monograph of *V. album* in the French Pharmacopeia [21]. Samples (10–20 μL/band) were applied as 10 mm bands with a 10 mm track distance onto the plate by a capillary 10 mm from the lower edge of the plate. TLC separations were achieved in a chamber (210 × 100 mm) saturated with distilled water:methanol:glacial acetic acid:dichloromethane at 2:3:8:15 (v/v/v/v) as a mobile phase. The bands were visualized under UV light (365 nm) before and after spraying with NP/PEG as the revealing solution.

### Determination of total flavonoid content

The concentration of total flavonoids as rutin equivalents was determined spectrophotometrically in the UV region (360 nm) in comparison with the standard curve of rutin absorption, adapted from Rolim et al. [22]. The concentration range of the standard curve comprised the following concentrations of rutin: 5.0, 10.0, 15.0, 20.0, 25.0 and 30.0 μg/mL. The absorptions of rutin concentration series were plotted to provide a linear calibration curve with r^2^ close to 1. A mixture of 95% ethanol and 0.02 M acetic acid (99:1) was used as the solvent in all solution preparations. The readings were performed in triplicate.

### HPLC viscotoxin analysis

Before HPLC analysis, all mother tincture samples were purified by solid phase extraction (Bakerbond, carboxylic acid, wide pore SPE column) to separate the VT from mother tincture impurities. For this, the SPE column was previously washed with methanol and water and subsequently equilibrated with 5 mL of a 200 mM ammonium solution. Aliquots from 0.5 to 2 mL of each mother tincture were added to the column and the pH was adjusted to 7.0–7.5. The samples were eluted under vacuum, and 3 mL of water was used to rinse the samples. Finally, the samples were eluted with 5 mL of 0.4 M acetic acid and the HPLC methodology described by Schaller et al. [7] was used for VT quantification. This involved injecting crude acid extracts into the column without further clean up. Column Nucleosil C18 AB, 125 × 4 mm with guard column; eluent A 0.1% TFA in water; eluent B: 0.1% TFA in acetonitrile/water = 60/40. The gradient was as follows: 0–9.0 min, 38% B to 42% B; 9.0–9.5 min, 42% B to 50% B; 9.5–17.0 min, 50% B to 54% B; 17.0–18.0 min, 54% B to 70% B; 18.0–19.0 min 70% B; 19.0–19.2 min 70% B to 38% B; 19.2–21.0 min 38% B; flow rate: 1 mL/min; detection, UV at 210 nm. The VT eluted in the order B, A1, A2, A3 and the total viscotoxin content (μg/mL) was calculated as the sum of each isoform.

### LC-HRMS analysis

Samples were prepared as described in the *V. album* monograph with modifications [21]. UHPLC Dionex Ultimate 3000 coupled to a Q Exactive Plus Orbitrap mass spectrometry system (Thermo Fisher Scientific, Germany), equipped with an electrospray ionization (ESI) source operating in negative ion mode at a voltage of 2.9 kV was employed for the chemical analysis. Separations were performed on a reversed-phase column (Thermo Syncronis C18 50 mm × 2.1 mm; 1.7 μm). Water-formic acid 0.1% v/v (A) and acetonitrile (B) were used as mobile phases as follows: (0–15 min, 2% B; 15–16.2 min, 20% B; 16.2–17 min, 100% B; 17–18 min, 100–2% B; 18–19.5 min 2% B). The flow rate was 0.4 mL/min and the injection volume was 5 μL. The mass spectra were acquired in full scan mode at a resolution of 70,000 over a *m/z* range of 60–780. Chromatograms were aligned using the XCMS online platform, and compound annotation was performed by comparing the exact mass and MS/MS spectra of compounds (Supplementary information) with those available in different databases (European MassBank, MassBank of North America – Mona, SciFinder Scholar, NIST MSMS 2014) and in the literature for *V. album*. A 5 ppm tolerance error between the theoretical and experimental mass values was considered for compound annotation.

### Cell lines and culture conditions

The following cell lines were used: MOLT-4 (human acute lymphoblastic leukemia cell line), Yoshida (mouse sarcoma cell line) and non-tumor NIH/3 T3 (mouse embryonic fibroblasts cell line), obtained from ATCC, Rockville, MD, USA (Yoshida and MOLT-4) and from the German Collection of Microorganisms and Cell Cultures GmbH, Braunschweig, Germany (NIH/3 T3). All cells were cultured in RPMI-1640 medium supplemented with 5% heat-inactivated fetal calf serum (FCS), 2 mM L-glutamine, and 1% penicillin–streptomycin in a humidified atmosphere with 5% CO_2_ at 37 °C. Cell lines were maintained in exponential growth, and cells from sub-confluent monolayers (Yoshida and NIH/3 T3) were harvested by trypsin-EDTA to carry out the experiments.

### Cell viability assay

Cell viability was evaluated by the WST-1 colorimetric methodology. Briefly, 90 μL of each cellular suspension containing 5 × 10^4^ cells/mL was pre-cultured in 96-well plates. After 24 h, 90 μL of *V. album* mother tinctures, pre-diluted in cellular culture medium, were added at concentrations varying from 0.05 to 0.5% v/v. Cellular viability rates were measured after incubation for 4 and 24 h by the addition of 20 μL of WST-1 to each well. The absorption at 450 nm and 650 nm against a background control was measured after 3 h of incubation at 37 °C in the dark in a multiwell plate reader. Since ethanol was used as the solvent for the extraction of tinctures, its effect on cell viability was also evaluated using the same MT concentrations. The percentage of viable cells was calculated in relation to control cells (untreated and treated with ethanol solvent) using mean values from at least three independent experiments, and the calculation was performed in triplicate. The IC50 was calculated using GraphPad 5 Software.

### Determination of apoptosis/necrosis by flow cytometry

Molt-4 and Yoshida cells were seeded on 6-well plates at a concentration of 1 × 10^5^ cells/well. After 24 h, cells were treated with the following MT concentrations (% v/v) for 4 and 24 h: VAM (0.15 and 0.5), VAQ (0.15 and 0.5), VAU (0.35 and 0.5), VAA (0.05 and 0.5), and VAP (0.35 and 0.5). After treatments, the cellular suspension (Molt-4) and supernatant (Yoshida) were collected. Trypsin was added to the supernatants of the Yoshida cells. Then, apoptosis/necrosis was measured using the Annexin V-FITC Apoptosis Detection Kit I (BD Biosciences Pharmingen). Apoptotic cells: Annexin V-FITC positive and 7-AAD negative. Late apoptotic/necrotic cells: Annexin V-FITC positive and 7-AAD positive. Values are given as the percentage of total cells in relation to the ethanol control.

### Statistical analysis

In vitro experiments were performed at least three different times, and results were analyzed by ANOVA with Dunnett’s post hoc test using GraphPad 5. *P* values < 0.05 were considered statistically significant.

## Results and discussion

### Chemical analysis of *V. album* ethanolic extracts

TLC plates of ethanolic extracts showed orange-yellowish bands at 365 nm UV light after spraying with NP/PEG reagent, and this result is typical of flavonoid compounds. Each MT exhibited one fluorescent blue spot with a Rf (retention factor) value similar to that of the chlorogenic acid standard (Rf 0.60). According to ANSM [21], chlorogenic acid is a marker of *V. album* species, and its identification is important to assure the quality of the vegetal material. Łuczkiewics et al. [23] also identified chlorogenic acid in alcoholic extracts of *V. album*, corroborating the results found in this work.

### Determination of the total flavonoid content of the mother tincture

Table 1 shows the flavonoid concentration expressed as mg/g of plant fresh weight (mg/g fw) after ethanolic extraction. The highest concentration was detected in *V. album* growing on *Quercus robur* (9.67) and *Malus domestica* (6.30). Pietrzak et al. [24] observed an increase in the flavonoid content of *V. album* ssp. *abietis* extracted by a mixture of polar organic solvents with water. Additionally, Pietrzak et al. [25] determined that the flavonoid content in methanol extracts from *V. album* growing on different host trees ranged from 0.270 to 0.428 mg/g. The host species and the organ harvested influenced the chemical composition of mistletoe [8]. 
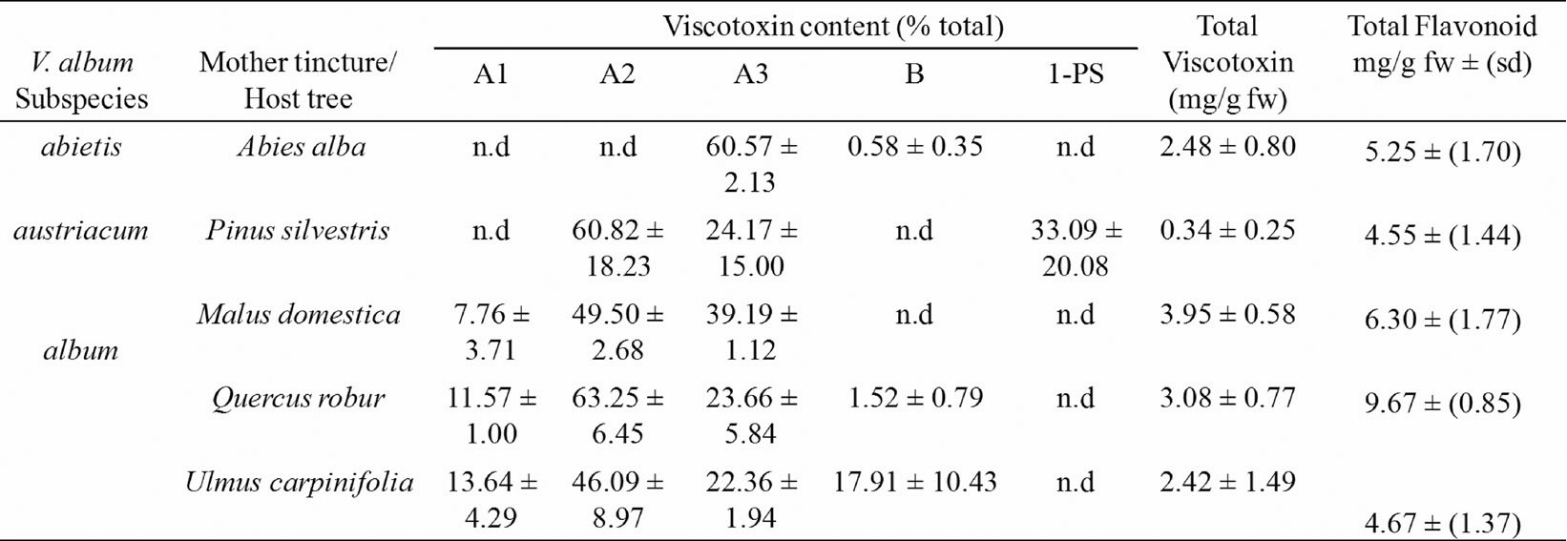

**Table 1:**
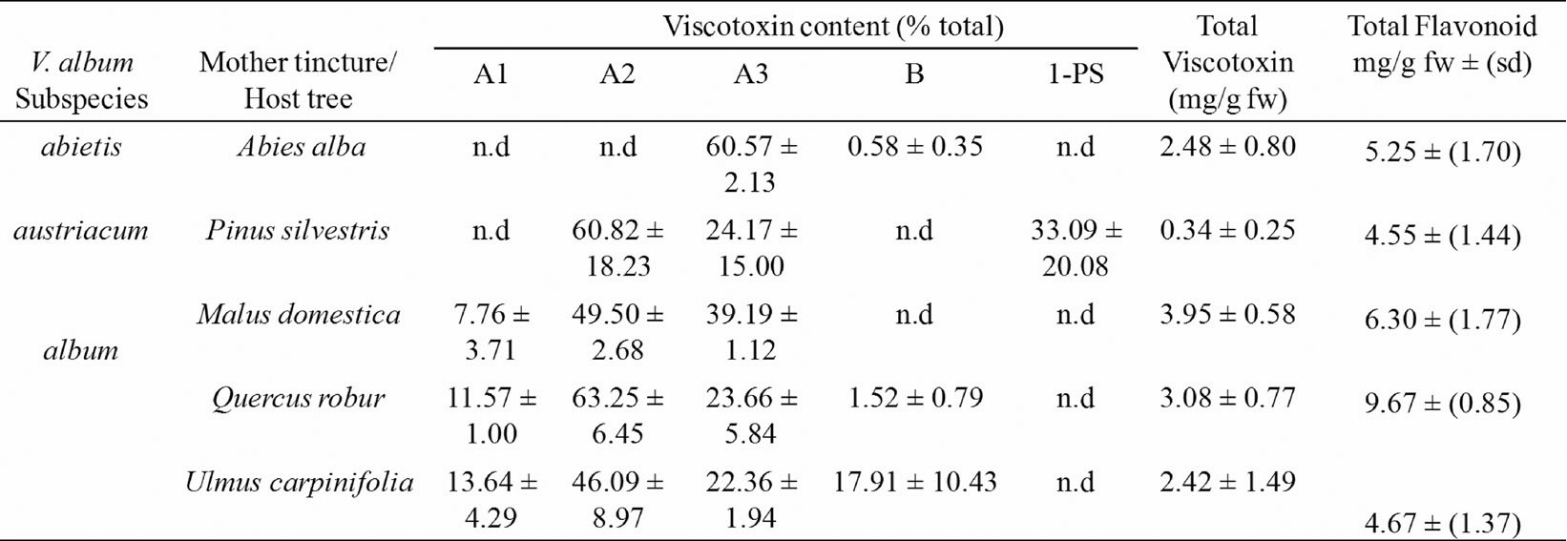
Total viscotoxin and flavonoid contents (mg/g fw) and proportions of the viscotoxin isoforms A1, A2, A3, B, and 1-PS in the *Viscum album* mother tincture samples, n.d: not detectable

### HPLC viscotoxin analysis

Quantitative analysis (Table 1) of VT isoforms A1, A2, A3, B and 1-PS in ethanolic extracts showed that *V. album* ssp. *abietis* contains predominantly viscotoxin A3, whereas *V. album* ssp. *austriacum* contains viscotoxins A2 and 1-PS, which were not detected in *V. album* ssp. *album*. The three European subspecies of *V. album* could be distinguished on the basis of their VT composition, and this result is in accordance with literature data [26, 27]. However, the total content of VT is greater in aqueous preparations than in hydroalcoholic preparations when the viscotoxin proportions are compared for the same *V. album* ssp. [27].

### LC-HRMS analysis

**Fig. 2:**
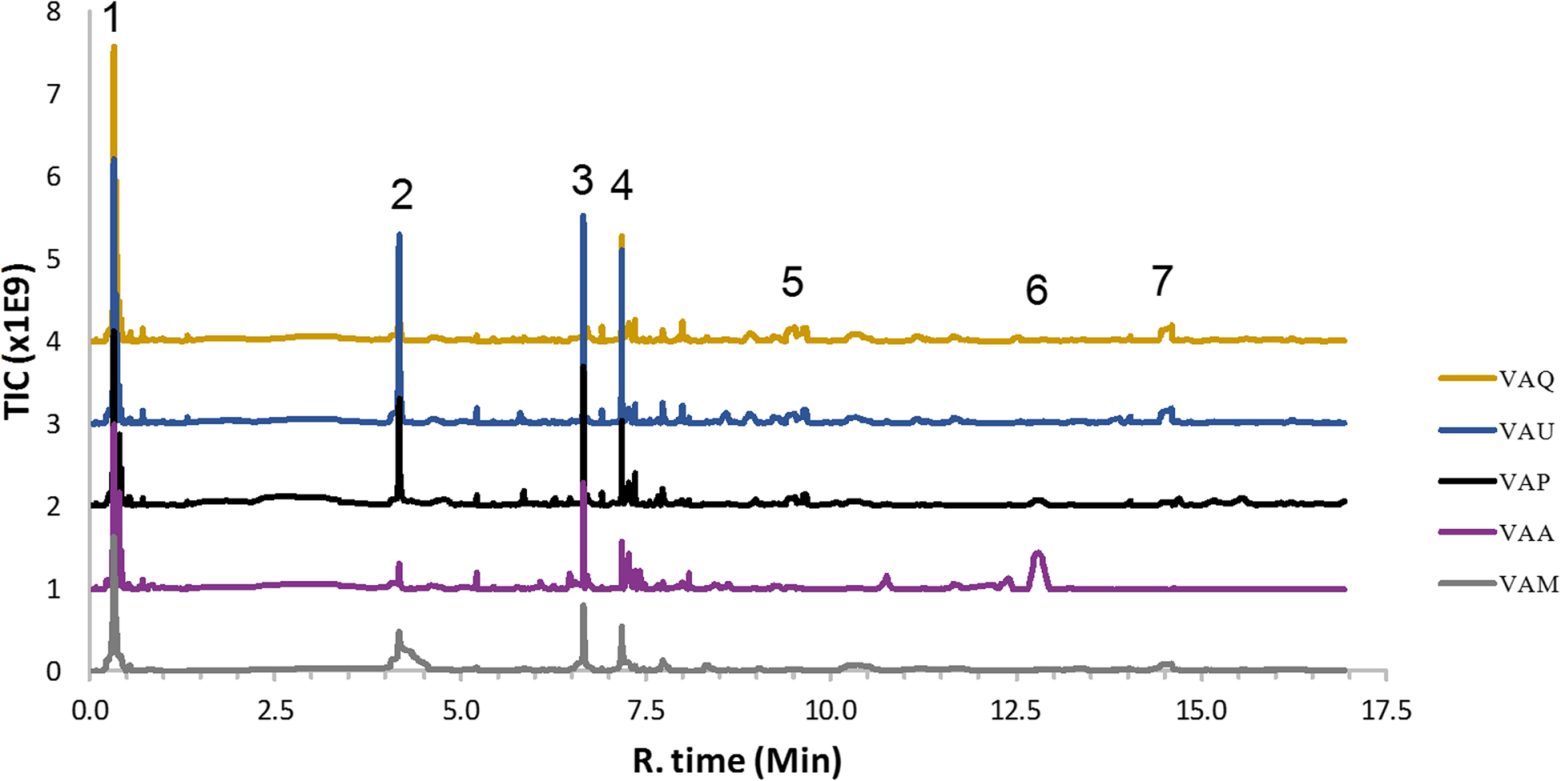
Total ion chromatogram (TIC) in negative electrospray ionization mode of the *Viscum album* mother tinctures analyzed by LC-HRMS. The abbreviations used are *V. album* ssp. *album* growing on *Malus domestica* (VAM), *Quercus robur* (VAQ) and *Ulmus carpinifolia* (VAU); *V. album* ssp. *abietis* growing on *Abies alba* (VAA); and *V. album* ssp. *austriacum* growing on *Pinus sylvestris* (VAP)


This analysis was performed to obtain an overview and compare the chemical compounds of the different *V. album* MTs prepared. The major peaks present in the LC-HRMS chromatograms were tentatively identified, and this compound identification may help in the standardization of the *V. album* extracts used in the in vitro analysis. Regarding the chemical complexity, a total of seven compounds (**1–7,** Fig. 2) were putatively annotated based on their highly accurate *m/z* values and MS/MS fragmentation spectra (see Supplementary material Fig. S1-S6). These peaks presented deprotonated molecular ions at *m/z*: 191.05585 [M-H]^−^ (C_7_H_11_O_6_), 353.08908 [M-H]^−^ (C_16_H_17_O_9_), 353.08908 [M-H]^−^ (C_16_H_17_O_9_), 353.08908 [M-H]^−^ (C_16_H_17_O_9_), 625.14038 [M-H]^−^ (C_27_H_29_O_17_), 565.15607 [M-H]^−^ (C_26_H_29_O_14_), and 581.22496 [M-H]^−^ (C_28_H_37_O_13_) (Table 2).

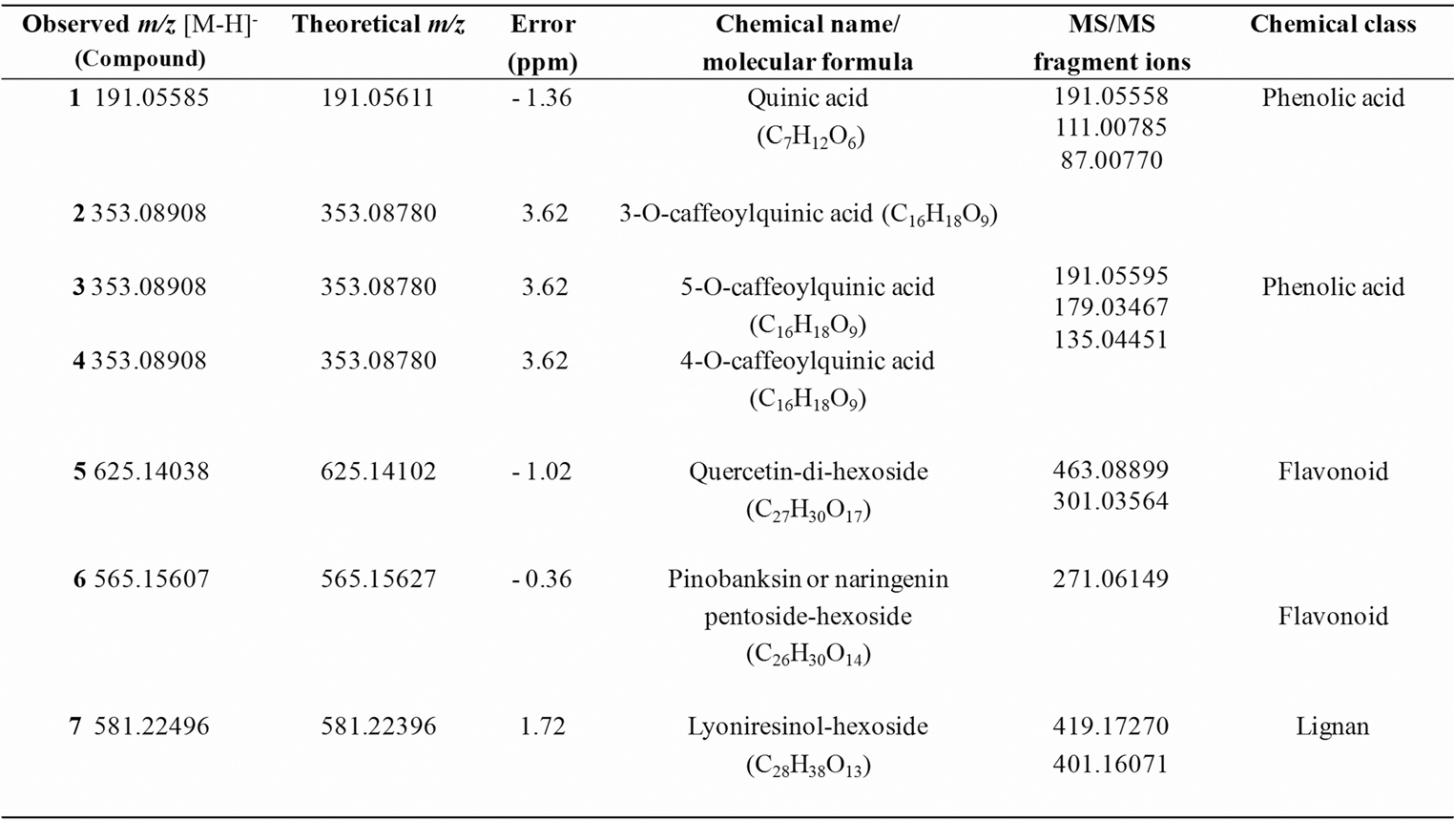

**Table 2:**
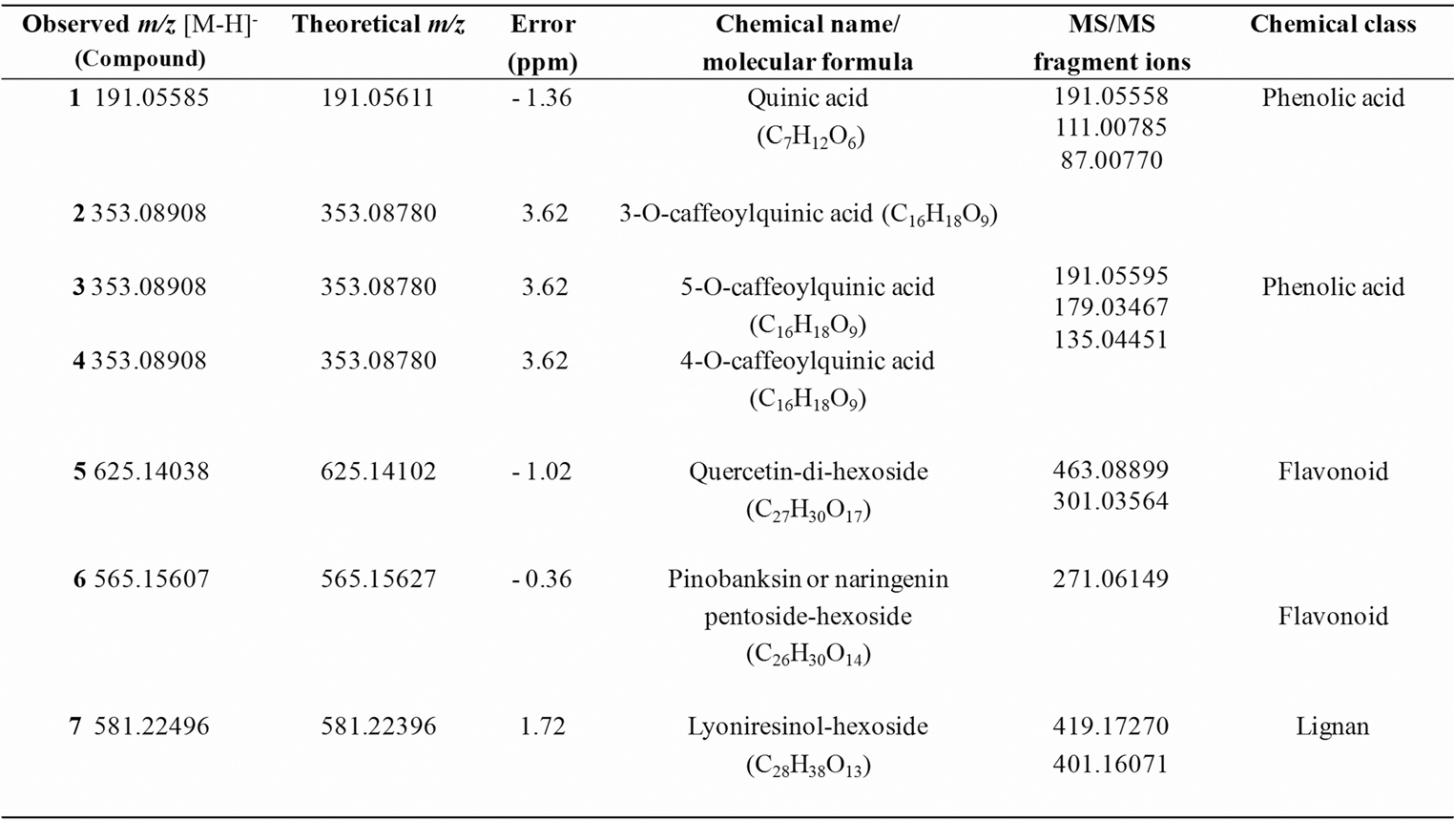
Chemical compounds putatively identified in *Viscum album* by LC-HRMS/MS in negative mode ionization

Compound **1** presented an ion at *m/z* 191.05585 [M-H]^−^, similar to quinic acid C_7_H_12_O_6_ (− 1.36 ppm error) [28]. Quinic acid has already been described in the alcoholic extract of *V. schimperi* [29].

Compounds **2, 3** and **4** were chlorogenic acid isomers and showed similar deprotonated molecular ions at *m/z* 353.08908 [M-H]^−^ in the following elution order: 3-O-caffeoylquinic acid, 5-*O*-caffeoylquinic acid and 4-*O*-caffeoylquinic acid, respectively (C_16_H_18_O_9_, 3.62 ppm error). This order is in accordance with Zhang et al. [30], who also used a reverse-phase C18 column. Chlorogenic acid has already been reported in the ethanol extract of *V. album* [16, 23] and is important to assure the quality of the vegetal material.

Compound **5** presented an ion at *m/z* at 625.14038 [M-H]^−^ and was tentatively identified as the flavonol quercetin-di-hexoside. The MS/MS spectrum of this compound showed a characteristic loss of two hexose units (M-162 Da).

Compound **6** showed an ion at *m/z* at 565.15607 [M-H]^−^ and a fragment ion at *m/z* 271 [M-H]^−^, which correspond to an aglycone moiety of a flavonoid structure, and two sugar unit losses: hexose (M-162 Da) and pentose (M-132 Da). Thus, compound **6** was identified as pinobanksin or naringenin pentoside-hexoside (C_26_H_30_O_14,_ 0.36 ppm error) [31–33]. This compound showed a higher intensity signal in VAA compared to the other ethanolic extracts analyzed.

Compound **7** presented a deprotonated molecular ion peak at *m/z* 581.22496 [M-H]^−^ with a fragment ion at *m/z* 419.17270, suggesting the loss of hexose. These compounds, already described in the genus *Viscum* [34], were assigned as lyoniresinol-hexoside (C_28_H_38_O_13_), with a − 1.72 ppm error [35].

### Cell viability assay

**Fig. 3:**
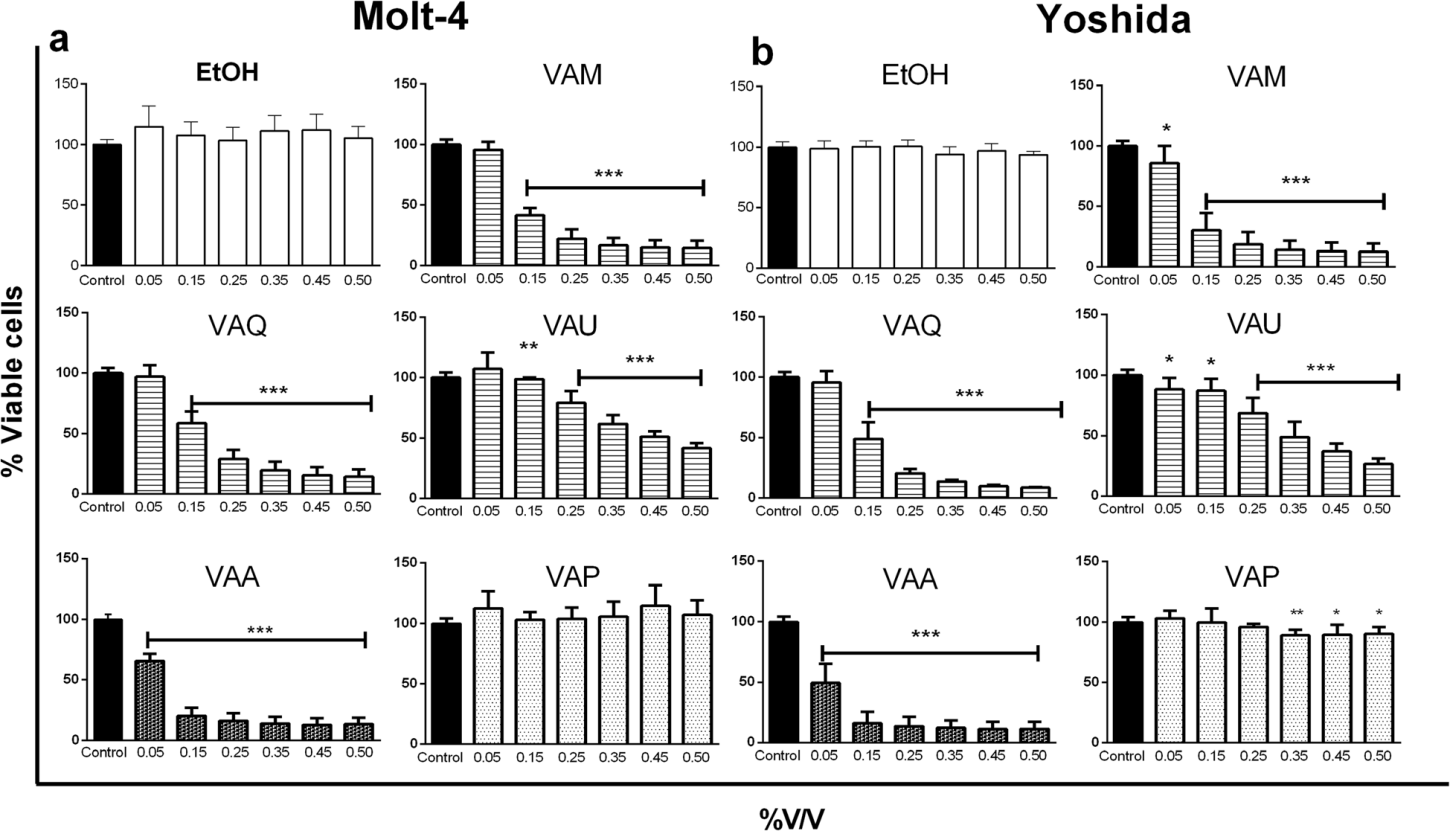
Dose response effect of five *Viscum album* mother tinctures on the proliferation of Molt-4 (**a**) and Yoshida cell lines (**b**) after 24 h. The final concentrations varied between 0.5 and 0.05% v/v. Cell growth kinetics were assessed with the WST-1 assay. The results are presented as the mean ± SD from three independent experiments in relation to control cells. **p < 0.001; ***p < 0.0001, obtained with one-way ANOVA with Dunnet’s post hoc test. Legend symbols: EtOH, cells incubated with ethanol solvent; Control, untreated cells; *V. album* ssp. *album* growing on *Malus domestica* (VAM), *Quercus robur* (VAQ) and *Ulmus carpinifolia* (VAU); *V. album* ssp. *abietis* growing on *Abies alba* (VAA); and *V. album* ssp. *austriacum* growing on *Pinus sylvestris* (VAP)

The toxicity of the alcoholic vehicle was first evaluated by WST assay in Molt-4 and Yoshida tumor cells, and no cellular toxicity was observed (Fig. 3a-b). Therefore, the next tests with MT were performed with vehicle concentrations between 0.05 and 0.5% v/v.

Molt-4 (Fig. 3a) and Yoshida cells (Fig. 3b) exhibited approximately the same sensitivity to the tinctures, with MT from *Abies alba* (VAA) being the most effective, with IC50 values of 0.07 ± 0.01% v/v and 0.05 ± 0.03% v/v for Molt-4 and Yoshida, respectively. The non-tumor cells (3 T3) were less sensitive with IC50 values of 1.60 ± 0.48% v/v after incubation with VAA. VAP did not exhibit toxicity in Molt-4 cells at any concentration tested (Fig. 3a) and exhibited only slight toxicity in Yoshida cells (*p* < 0.05; concentration range from 0.35–0.50, Fig. 3b).

**Fig. 4:**
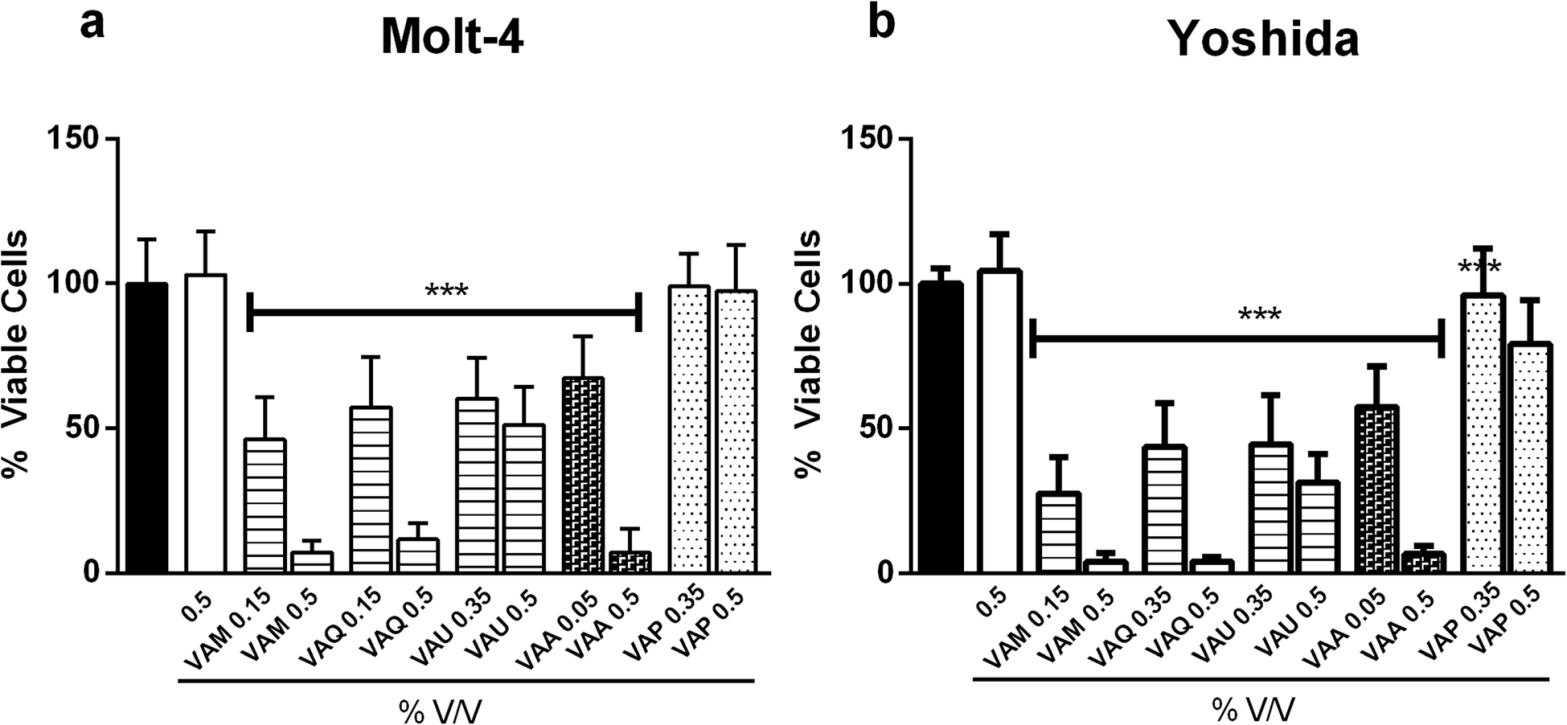
WST-1 assay after 4 h of incubation with different concentrations of five *Viscum album* mother tinctures (VAM, *V. album* ssp. *album* growing on *Malus domestica;* VAQ, *V. album* ssp. *album* growing on *Quercus robur;* VAU, *V. album* ssp. *album* growing on *Ulmus carpinifolia;* VAA, *V. album* ssp. *abietis* growing on *Abies alba;* and VAP, *V. album* ssp. *austriacum* growing on *Pinus sylvestris*). **a** Molt-4 and **b** Yoshida cell lines. The results are presented as the mean ± SD from three independent experiments in relation to control cells. ***p < 0.0001, obtained with one-way ANOVA with Dunnet’s post hoc test. Legend symbols: EtOH, cells incubated with ethanol solvent; Control, untreated cells; *V. album* ssp. *album* growing on *Malus domestica* (VAM), *Quercus robur* (VAQ) and *Ulmus carpinifolia* (VAU); *V. album* ssp. *abietis* growing on *Abies alba* (VAA); and *V. album* ssp. *austriacum* growing on *Pinus sylvestris* (VAP)

The anti-proliferative assay highlighted the antitumor potential of MT within 4 h of incubation (Fig. 4a-b), in which a significant reduction in viability of approximately 90% was detected (0.5% v/v VAA, VAM, VAQ; *p* < 0.0001).

**Fig. 5:**
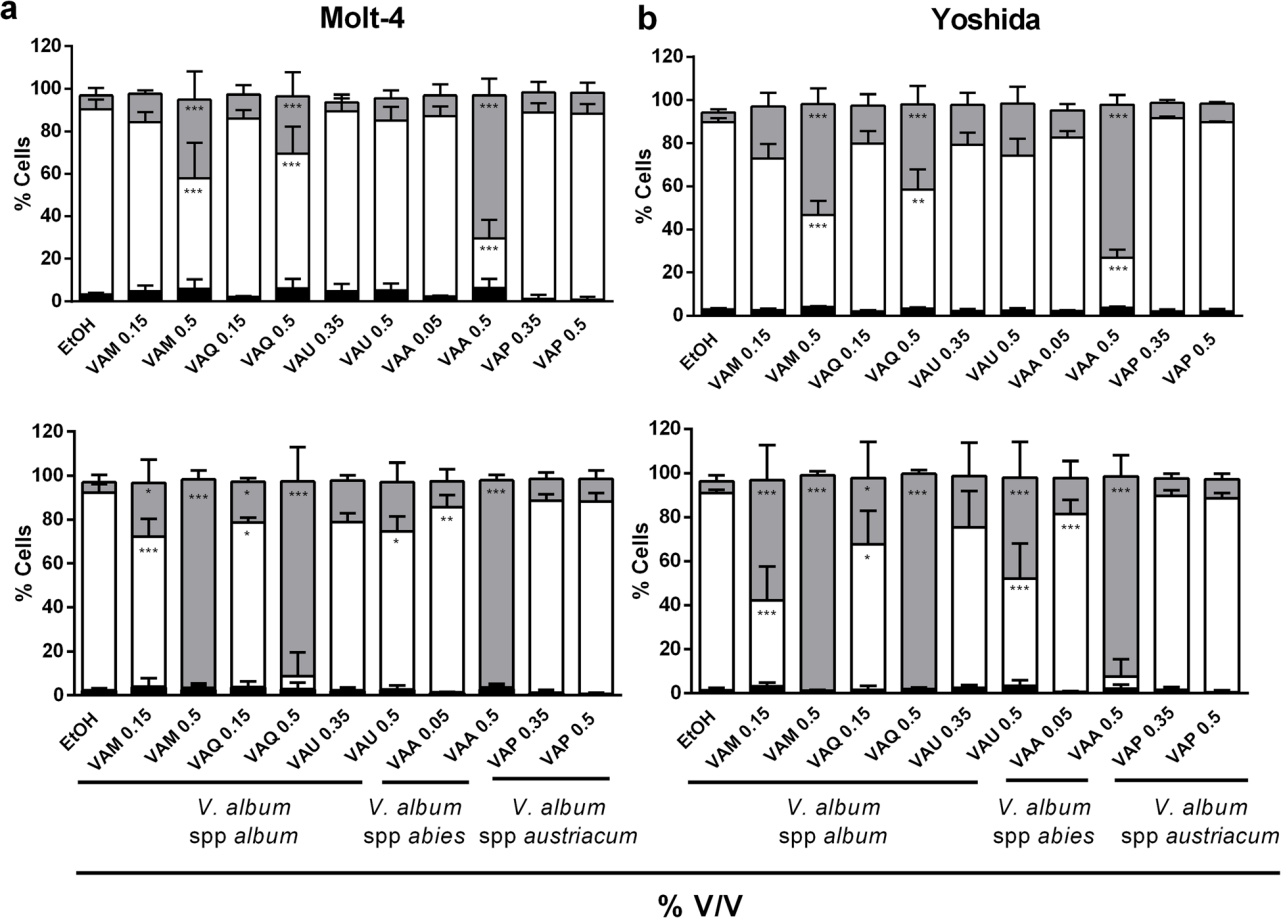
Flow cytometry analyses of Molt 4 (**a**) and Yoshida (**b**) cell lines. Mean values (± SD) of apoptotic, necrotic  and viable cells (white bar) after 4 h (top) and 24 h (bottom) of incubation with different concentrations of five *Viscum album* mother tinctures (VAM, *V. album* ssp. *album* growing on *Malus domestica;* VAQ, *V. album* ssp. *album* growing on *Quercus robur;* VAU, *V. album* ssp. *album* growing on *Ulmus carpinifolia;* VAA, *V. album* ssp. *abietis* growing on *Abies alba;* VAP, *V. album* ssp. *austriacum* growing on *Pinus sylvestris*). The results are presented as the mean ± SD from four independent experiments. *p < 0.05; **p < 0.001; ***p < 0.0001 obtained with one-way ANOVA with Dunnett’s post hoc test

Flow cytometry was used to characterize the mechanism of cell death induced by *V. album* MT after 4 and 24 h of incubation (Fig. 5a-b). The number of necrotic cells increased proportionally to the MT concentration and to the incubation time, with a very similar profile for both cell lines, except in response to VAP. In Molt-4 cells, 0.5% v/v VAA, VAM and VAQ induced 61, 31 and 21% necrotic cell death, respectively, after 4 h of incubation (Fig. 5a top). Similar percentages of necrosis were observed in Yoshida cells (66, 47 and 35%), respectively, with the same MT concentrations. The necrotic effects were more evident after 24 h, supporting the necrotic potential of *Abietis*, *Malus* and *Quercus* MT (Fig. 5b bottom).

The present data suggest that the antitumor activity of MT from *V. album* ssp. *abietis* may be related to its higher content of VT A3 in addition to the presence of small molecules, as discussed below. Indeed, Schaller et al. [26] demonstrated that the viscotoxin A3 of *V. album* aqueous extract was more cytotoxic than other VT isoforms when the Yoshida sarcoma cell line was evaluated [26]. Additionally, Coulon et al. [36] attributed the highest antitumor activity of viscotoxin A3 to its physicochemical characteristics, in which the hydrophobic residues and the net liquid charge were able to increase the cell membrane interaction.

In addition, in vitro studies revealed that methanol extracts of mistletoe berries and ethanolic tinctures from whole *V. album* decreased the proliferation of colon cancer cells and viability of the murine melanoma lineage in a dose-dependent manner, respectively [16, 25]. These cellular alterations were attributed to modifications of mitochondrial activity and the cell cycle, among other cellular damage. Both studies showed that the polyphenolic composition of these *V. album* extracts was involved in the antitumor activity.

Regarding the seven compounds tentatively identified in this study, compound 1 was able to promote cytotoxicity in squamous cell carcinoma 4 (SCC-4). Compounds 2, 3 and 4 were chlorogenic acid isomers formed by quinic and cinnamic acid esterification [37]. Recently, the antitumor potential of chlorogenic acid was described in in vitro and in vivo models [38, 39]. Additionally, Pan et al. [40] demonstrated that a type of quercetin-di-hexoside (quercetin 3,4′-di-*O*-glucoside) was an effective inhibitor of the growth of HepG2, PC3 and HT29 cells, corroborating the antitumor potential of *V. album* ethanolic extracts.

Pinobanksin or naringenin pentoside-hexoside (compound 6) presented a higher peak intensity in *V. album* extracts prepared with *V. album* ssp. *abietis* when compared to the other ethanolic extracts. The antitumor potential of these flavanones was previously described in tumor cell lines [41, 42]. In addition, the metabolomics analyses performed by our group with 50 different *V. album* samples harvested in winter and summer seasons from the same habitat in Switzerland also confirmed the importance of compound 6 in subspecies sample differentiation (*data not shown*).

Compound 7 was identified as lyoniresinol-hexoside, and its aglycone has already been described in *V. album* ssp. *coloratum* [35]. This lignan had cytotoxic properties against B16F10 cells after 48 h of treatment [43]. In addition, Baek et al. [44] demonstrated the ability of (−)-9′-*O*-(α-L-Rhamnopyranosyl) lyoniresinol to suppress A2780 human ovarian carcinoma cell proliferation in a dose-dependent manner.

## Conclusion

The present study shows the antitumor potential of *V. album* tinctures in in vitro models. The cell death mechanism involved necrotic effects depending on the influence of the host tree, time and dose. Mother tincture from *V. album* ssp. *abietis* growing on *Abies alba* was the most effective, probably because of the higher content of VT A3, while MT from *V. album* ssp. *austriacum* growing on *Pinus sylvestris* exhibited only a slight antitumor effect. Additionally, tumor cells were more sensitive than normal fibroblasts, suggesting that *V. album* MT has promising antitumor potential. The small molecules identified in MT were quinic acid, three isomers of chlorogenic acid, quercetin-di-hexoside, pinobankasin or naringenin pentoside-hexoside and lyoniresinol-hexoside. Further studies using in vitro and in vivo models in addition to stability assessments, should be performed to stimulate the development of new pharmaceutical formulations containing *V. album* ethanolic extracts.

## Supplementary information

**Additional file 1.**

## Data Availability

The datasets used and/or analyzed during the current study are available from the corresponding author on reasonable request.

## Abbreviations

EDTA: Ethylenediaminetetraacetic acid
ESI: Electrospray ionisation
FCS: Fetal calf serum
HPLC: High-performance liquid chromatography
HRMS: High-resolution mass spectrometry
IC50: Half maximal inhibitory concentration
LC-HRMS: Liquid chromatography-high resolution mass spectrometry
MS: Mass spectrometry
MT: Mother tincture
NP/PEG: Diphenylboriloxyethilamine/polyetileneglicol
PDA: Photodiode array detector
Rf: Retention factor
TIC: Total ion current
TLC: Thin layer chromatography
VAA: *V. album* from *Abies alba*
*V. album*: *Viscum album*
VAP: *V. album* from *Pinus sylvestris*
VAM: *V. album* from *Malus domestica*
VAQ: *V. album* from *Quercus robur*
VAU: *V. album* from *Ulmus carpinifolia*
VT: Viscotoxin
WST-1: [2-(4-Iodophenyl)-3-(4-nitrophenyl)- 5-(2,4-disulfophenyl)-2H-tetrazolium]
% v/v: % volume/volume
