# Supplementary material for: Phytochemical analysis and in vitro anti-proliferative activity of Viscum album ethanolic extracts
Source: BMC Complement Med Ther. 2020 Jul 9;20:215. doi: 10.1186/s12906-020-02987-4 (PMC7346636; doi:10.1186/s12906-020-02987-4)
Supplement: Supplementary file 1 — Additional file 1. [file 12906_2020_2987_MOESM1_ESM.pdf]

Supplementary Information

Phytochemical analysis and *in vitro* antiproliferative activity of *Viscum album* ethanolic extracts

Carla Holandino[1,4], Michelle Nonato de Oliveira Melo[1,3], Adriana Passos Oliveira[1], João Vitor da Costa Batista[1], Marcia Alves Marques Capella[2], Rafael Garrett[3], Mirio Grazi[4], Hartmut Ramm[4], Claudia Dalla Torre[4], Gerhard Schaller[4], Konrad Urech[4], Ulrike Weissenstein[4], Stephan Baumgartner[4,5,6]

[1]*Federal University of Rio de Janeiro, Multidisciplinary Laboratory of Pharmaceutical Sciences, Faculty of Pharmacy, Rio de Janeiro, Brazil*
[2]*Federal University of Rio de Janeiro, Biophysics Institute, Rio de Janeiro, Brazil*
[3] *Federal University of Rio de Janeiro, Metabolomics Laboratory, Chemistry Institute, Rio de Janeiro, Brazil*
[4]*Society for Cancer Research, Hiscia Institute, Arlesheim, Switzerland*
[5]*University of Bern, Institute of Complementary and Integrative Medicine, Bern, Switzerland*
[6]*University of Witten/Herdecke, Institute for Integrative Medicine, Herdecke, Germany*

V. album mother tinctures analysis by LC-HRMS/MS was performed using a phase-reverse C18 column (Hypersil Gold 100 mm x 2.1 mm; 3.0 μm particle size; Thermo Fisher Scientific). Mobile phase was: water-formic acid 0.1% v/v (A) and acetonitrile-formic acid 0.1% v/v (B). The elution gradient was: (i) 0-1 min, 10% B; (ii) 1-16 min, 10-95% B; iii) 16-18 min, 95% B; (iv) 18-18.1 min, 95-10% B; (v) 18.1-22 min 10% B at a flow rate of 350 µL/min and injection volume of 5 µL. Mass spectra were acquired in full scan mode in positive and negative-ESI over a range of *m/z* 100-1000 and resolution of 35.000 k followed by MS/MS fragmentation analysis (DDA ddMS2 top 3). Date were acquired and processed on the Xcalibur v.2.0.7 (Thermo Scientific, Bremen, Germany).

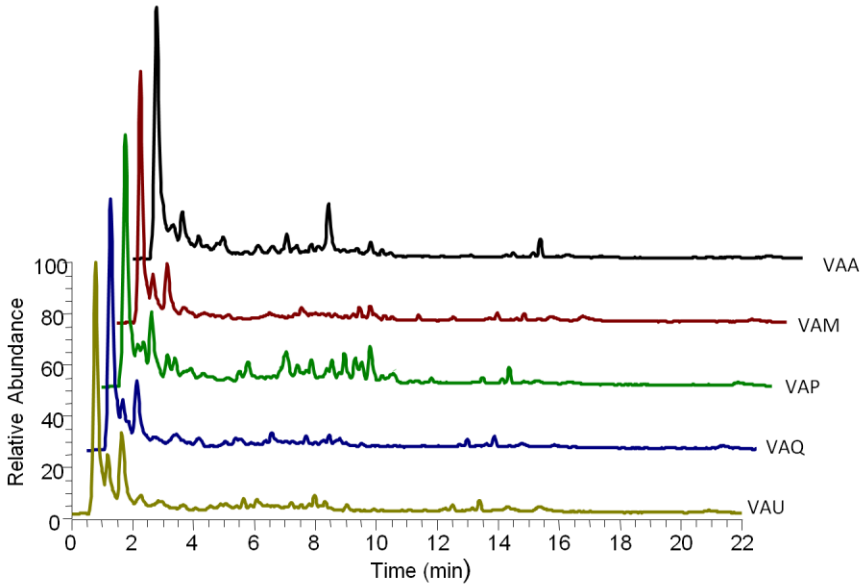

Figure S1. Total ion chromatogram (TIC) in negative electrospray ionization mode of the *Viscum album* mother tinctures analyzed by LC-HRMS. The abbreviations used are: *V. album* ssp. *album* growing on *Malus domestica* (VAM), *Quercus* robur (VAQ) and *Ulmus* carpinifolia (VAU); *V. album* ssp. *abietis* from *Abies alba* (VAA); *V. album* ssp. *austriacum* from *Pinus sylvestris* (VAP).

a) Precursor ion 191.05585 $[M-H]^-$ (compound 1)

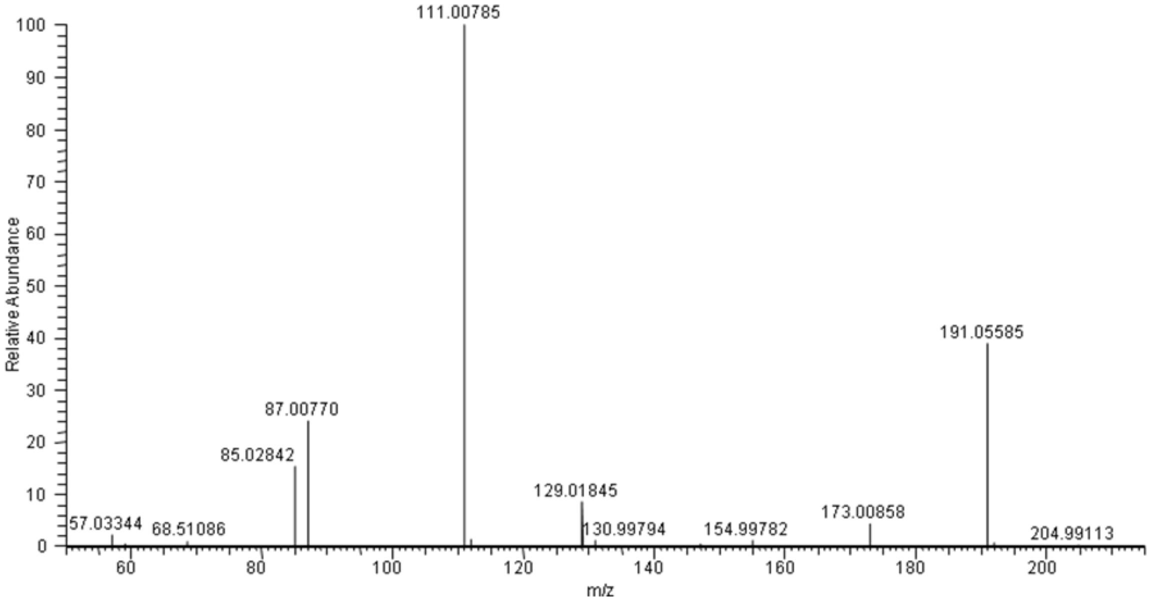

Figure S2: Mass fragmentation spectra obtained by LC-HRMS/MS of 191.05585 [M-H] putatively identified in *V. album* mother tinctures.

b) Precursor ion 353.08908 [M-H]$^-$ (compounds 2, 3 and 4)

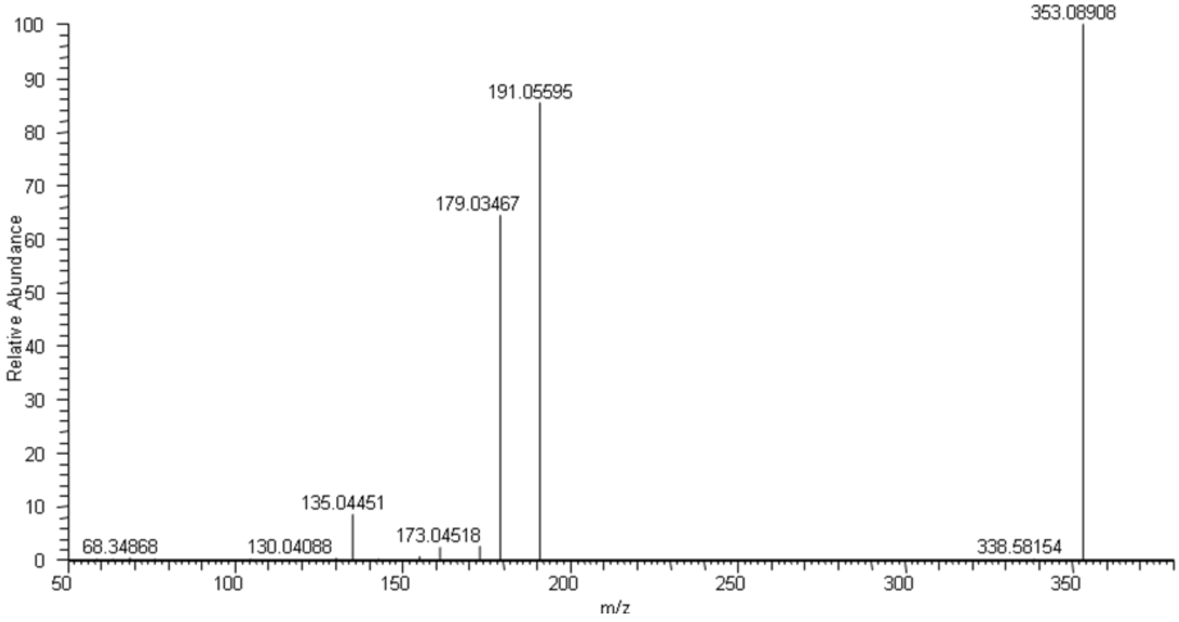

Figure S3: Mass fragmentation spectra obtained by LC-HRMS/MS of 353.08908 [M-H] putatively identified in *V. album* mother tinctures.

c) Precursor ion 625.14038 [M-H]$^-$ (compound 5)

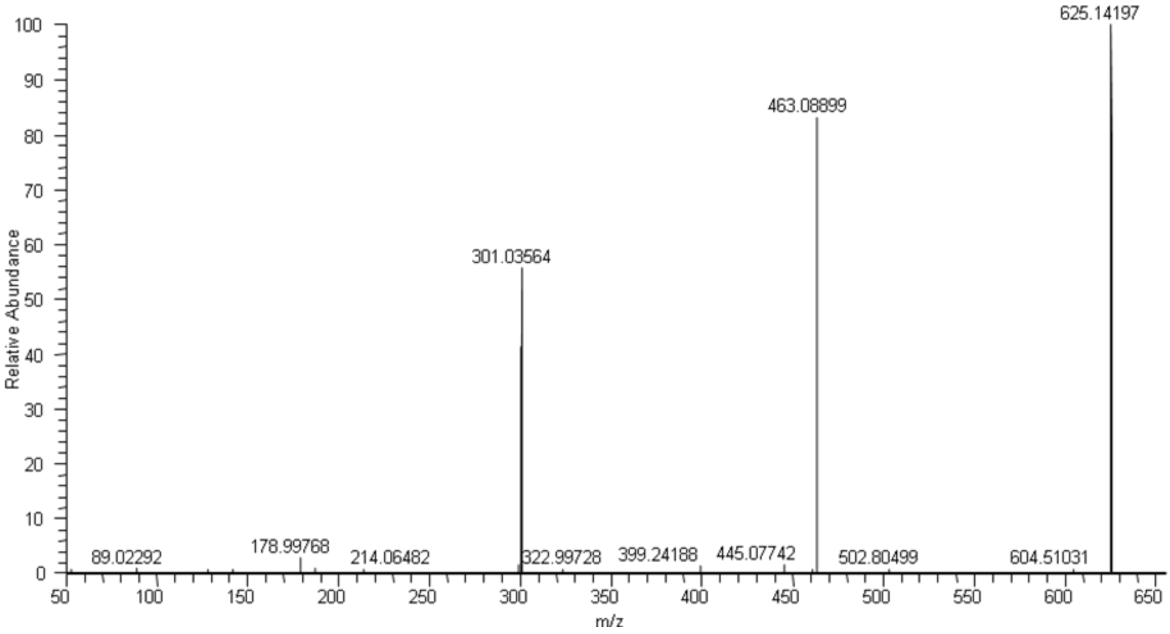

Figure S4: Mass fragmentation spectra obtained by LC-HRMS/MS of 625.14038 [M-H]$^-$ putatively identified in *V. album* mother tinctures.

d) Precursor ion 65.15607 [M-H]$^-$ (compound 6)

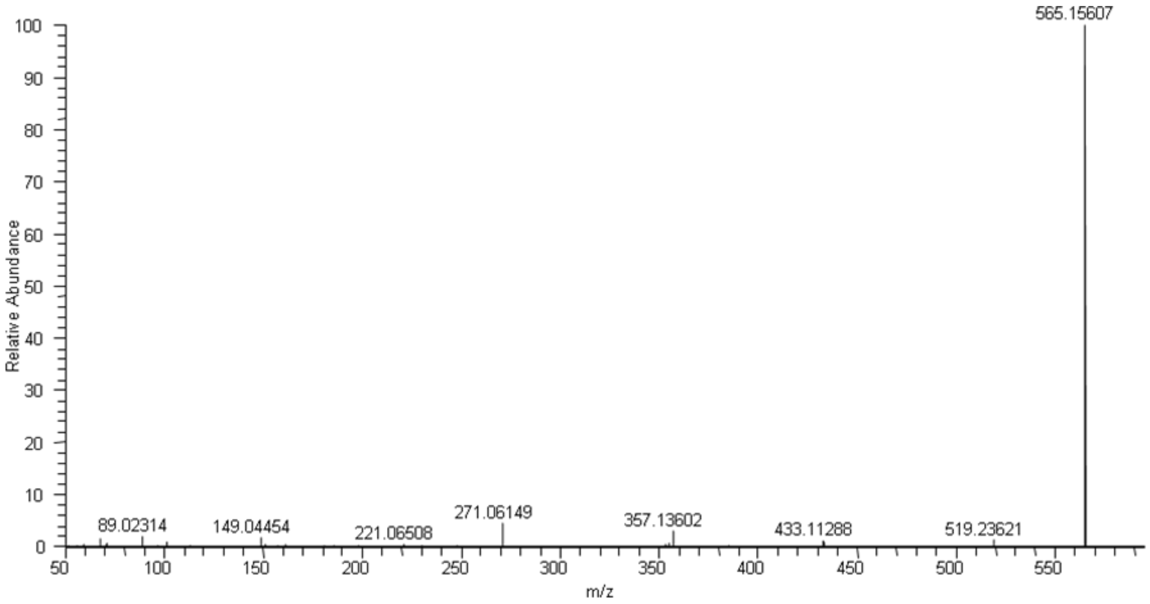

Figure S5: Mass fragmentation spectra obtained by LC-HRMS/MS of 565.15607 [M-H] putatively identified in *V. album* mother tinctures.

e) Precursor ion 581.22498 [M-H]$^-$ (compound 7)

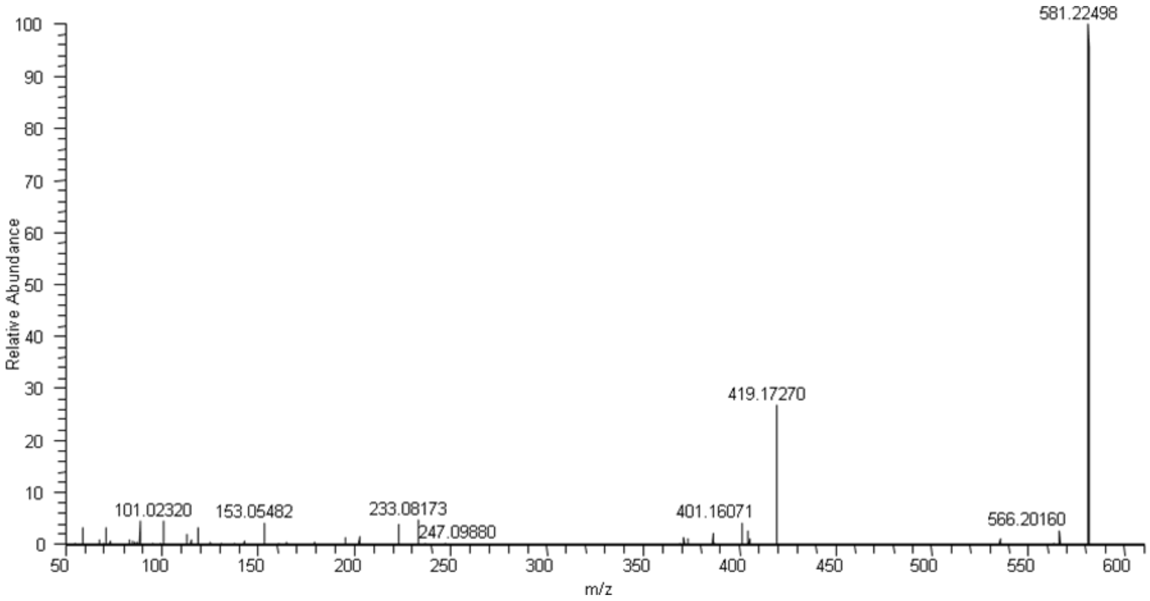

Figure S6: Mass fragmentation spectra obtained by LC-HRMS/MS of 581.22498 [M-H] putatively identified in *V. album* mother tinctures.
